# Supplementary material for: Whole blood RNA extraction efficiency contributes to variability in RNA sequencing data sets
Source: PLoS One. 2023 Nov 16;18(11):e0291209. doi: 10.1371/journal.pone.0291209 (PMC10653446; doi:10.1371/journal.pone.0291209)
Supplement: S2 File — Summary of the experimental rationale and ANOVA statistical analysis. (DOCX) [file pone.0291209.s002.docx]

S3: Normalization and Statistical Analysis

**TPM Count Normalization of 8721 Common mRNA Transcripts in Three Archived Data Sets.**

Before initiating the analysis of the three archived files, we evaluated the within-file means of 8721 common transcripts to establish our normalization approach. The within-file means of the mRNA transcripts in the C9, C12 and C35 data sets were 21.91 ± 129, 23.28 ± 111, and 9.79 ± 57, respectively. Single Factor ANOVA of the within-file means identified significant differences (F=44.7, P< 0.005). Furthermore, Student-Newman-Keuls’ range test revealed that the mean of the C35 data set was significantly smaller than either the C9 or C12 data set means (9.79, 21.91, 23.28, P < 0.5). Additional analysis of the three files revealed that these differences were likely due to number of total TPM counts within the different data sets (e.g., C9= 191,114, C12= 203,020 and C35= 85,374) and after normalizing the transcripts to an equivalent number of 200,000 total counts, the computed means were identical for all three data sets (22.93) with calculated CV of 5.89, 4.79 and 5.78 in the C9, C12 and C35 data sets, respectively. Although the CV values are remarkably high, this variance is expected since the transcript expression levels range from 0.151 (C9, EGF) to values of 8987 (C35, B2M) TPM counts.

**Calculation of the Slope and Intercept of the fifty-six samples employed in this study.**

Slope and intercept analysis of the fifty-six individual samples was undertaken to determine if either TPM count normalization or the selection of similar transcripts within the three data files has a significant influence on the regression analysis depicted in Figures 4 and 5 of the manuscript. For this analysis, the total number of mRNA and lncRNA transcripts with TPM counts > 0.1 and known size measurements were used to compute slope and intercept values for the samples. The individual computed slopes and intercepts for the samples are depicted below.

|  | Slope Calculations | | |  | Intercept calculations | | |
| --- | --- | --- | --- | --- | --- | --- | --- |
|  |  |  |  |  |  |  |  |
| Sample | C9 | C12 | C35 |  | C9 | C12 | C35 |
| 1 | -0.6884 | -0.7378 | 0.0693 |  | 2.7737 | 3.4121 | 0.0866 |
| 2 | -0.5263 | -0.696 | 0.0141 |  | 2.2902 | 3.2906 | 0.1877 |
| 3 | -0.7121 | -0.6838 | 0.0835 |  | 2.9014 | 3.1809 | -0.0512 |
| 4 | -0.7008 | -0.6139 | 0.059 |  | 2.8831 | 3.0069 | -0.0606 |
| 5 | -0.8659 | -0.564 | 0.1251 |  | 3.3881 | 2.8915 | -0.1072 |
| 6 | -0.5654 | -0.7669 | 0.0834 |  | 2.4142 | 3.5152 | -0.1351 |
| 7 | -0.6992 | -0.664 | 0.0367 |  | 2.8102 | 3.1931 | 0.1838 |
| 8 | -0.1772 | -0.767 | 0.1398 |  | 1.2104 | 3.5443 | -0.241 |
| 9 | -0.4656 | -0.6689 | 0.1225 |  | 2.1806 | 3.1693 | -0.0918 |
| 10 |  | -0.5695 | 0.093 |  |  | 2.9083 | -0.0334 |
| 11 |  | -0.5226 | 0.0889 |  |  | 2.7458 | 0.0086 |
| 12 |  |  | 0.0582 |  |  | 2.8435 | 0.0849 |
| 13 |  |  | 0.0934 |  |  |  | -0.0327 |
| 14 |  |  | 0.1095 |  |  |  | -0.0874 |
| 15 |  |  | 0.1308 |  |  |  | -0.0939 |
| 16 |  |  | 0.0844 |  |  |  | -0.0715 |
| 17 |  |  | 0.061 |  |  |  | -0.1018 |
| 18 |  |  | 0.1262 |  |  |  | -0.1673 |
| 19 |  |  | 0.1121 |  |  |  | -0.1541 |
| 20 |  |  | 0.0757 |  |  |  | -0.0232 |
| 21 |  |  | 0.0925 |  |  |  | -0.2038 |
| 22 |  |  | 0.1461 |  |  |  | -0.2136 |
| 23 |  |  | 0.1171 |  |  |  | -0.1411 |
| 24 |  |  | 0.1606 |  |  |  | -0.295 |
| 25 |  |  | 0.1239 |  |  |  | -0.2005 |
| 26 |  |  | 0.1659 |  |  |  | -0.2254 |
| 27 |  |  | 0.1656 |  |  |  | -0.3041 |
| 28 |  |  | 0.0292 |  |  |  | -0.0291 |
| 29 |  |  | 0.0906 |  |  |  | -0.0548 |
| 30 |  |  | 0.0826 |  |  |  | -0.058 |
| 31 |  |  | 0.0991 |  |  |  | -0.1253 |
| 32 |  |  | 0.1019 |  |  |  | -0.1257 |
| 33 |  |  | 0.0852 |  |  |  | -0.1326 |
| 34 |  |  | 0.0208 |  |  |  | 0.2107 |
| 35 |  |  | 0.1123 |  |  |  | -0.1098 |

The Microsoft Excel Analysis ToolPak (version 2304, 16327.20248) was used to perform one-way ANOVA on the slope and intercept values from the three data sets and the results are listed below.

| ANOVA: Single Factor | |  |  |  |  |  |
| --- | --- | --- | --- | --- | --- | --- |
|  |  |  |  |  |  |  |
| SUMMARY |  |  |  |  |  |  |
| *Groups* | *Count* | *Sum* | *Average* | *Variance* |  |  |
| Slope | 35 | 3.36 | 0.096 | 0.001480049 |  |  |
| Slope | 12 | -7.8042 | -0.6503 | 0.007302172 |  |  |
| Slope | 9 | -5.4009 | -0.6001 | 0.039317127 |  |  |
|  |  |  |  |  |  |  |
|  |  |  |  |  |  |  |
| ANOVA |  |  |  |  |  |  |
| *Source of Variation* | *SS* | *df* | *MS* | *F* | *P-value* | *F crit* |
| Between Groups | 6.90828024 | 2 | 3.45414 | 411.2232568 | 5.3E-33 | 3.171626 |
| Within Groups | 0.44518257 | 53 | 0.0084 |  |  |  |
|  |  |  |  |  |  |  |
| Total | 7.35346281 | 55 |  |  |  |  |

| ANOVA: Single Factor | |  |  |  |  |  |
| --- | --- | --- | --- | --- | --- | --- |
|  |  |  |  |  |  |  |
| SUMMARY |  |  |  |  |  |  |
| *Groups* | *Count* | *Sum* | *Average* | *Variance* |  |  |
| Intercept | 35 | -2.9087 | -0.08311 | 0.015333 |  |  |
| Intercept | 12 | 37.7015 | 3.141792 | 0.071157 |  |  |
| Intercept | 9 | 22.8519 | 2.5391 | 0.383806 |  |  |
|  |  |  |  |  |  |  |
|  |  |  |  |  |  |  |
| ANOVA |  |  |  |  |  |  |
| *Source of Variation* | *SS* | *df* | *MS* | *F* | *P-value* | *F crit* |
| Between Groups | 117.3775432 | 2 | 58.68877 | 711.0543 | 5.24E-39 | 3.171626 |
| Within Groups | 4.374496908 | 53 | 0.082538 |  |  |  |
|  |  |  |  |  |  |  |
| Total | 121.7520401 | 55 |  |  |  |  |

The slope and intercept variance are markedly different between the three data sets as illustrated in the above ANOVA tables, varying by a relative slope ratio of 26.6: 4.93: 1 and intercept ratio of 25.0: 4.64: 1, respectively in the C9, C12 and C35 data sets. Due to major differences in variation, the nonparametric Kruskal Wallis test was also employed to test for significant differences. Analysis with either parametric or nonparametric tests demonstrate highly significant differences between the slope and intercept of the C35 data set and the slope and intercepts of the C9 and C12 data sets.

| Slope Kruskal Wallis Test H statistic is 38.6908 (2, n=56 (P is < 0.00001)) |
| --- |
| Intercept Kruskal Wallis Test H statistic is 39.7398 (2, n=56 (P is < 0.00001)) |
